# Oncogenes, Proto-Oncogenes, and Lineage Restriction of Cancer Stem Cells

**DOI:** 10.3390/ijms22189667

**Published:** 2021-09-07

**Authors:** Geoffrey Brown

**Affiliations:** Institute of Clinical Sciences, School of Biomedical Sciences, College of Medical and Dental Sciences, University of Birmingham, Edgbaston, Birmingham B15 2TT, UK; g.brown@bham.ac.uk; Tel.: +44-(0)121-414-4082

**Keywords:** stem cells, oncogenes, proto-oncogenes, lineage determination, epigenetics

## Abstract

In principle, an oncogene is a cellular gene (proto-oncogene) that is dysfunctional, due to mutation and fusion with another gene or overexpression. Generally, oncogenes are viewed as deregulating cell proliferation or suppressing apoptosis in driving cancer. The cancer stem cell theory states that most, if not all, cancers are a hierarchy of cells that arises from a transformed tissue-specific stem cell. These normal counterparts generate various cell types of a tissue, which adds a new dimension to how oncogenes might lead to the anarchic behavior of cancer cells. It is that stem cells, such as hematopoietic stem cells, replenish mature cell types to meet the demands of an organism. Some oncogenes appear to deregulate this homeostatic process by restricting leukemia stem cells to a single cell lineage. This review examines whether cancer is a legacy of stem cells that lose their inherent versatility, the extent that proto-oncogenes play a role in cell lineage determination, and the role that epigenetic events play in regulating cell fate and tumorigenesis.

## 1. Introduction

There are more than 200 types of cancer that are documented according to where they first develop and their similarity to a histological cell type within the tissue of origin. In this case, cancers may be viewed as an abundance of cells that resemble one partially mature or mature tissue cell type. The cancer stem cell theory states that the hierarchy of cells within a cancer is sustained by cancer stem cells (CSCs) [1]. They arise in a tissue-specific stem or progenitor cell that is able to give rise to the different types of functional cells within a tissue. It is well-known that chronic myeloid leukemia (CML) originates in a hematopoietic stem cell (HSC) [2], but paradoxically the offspring of the leukemia stem cells (LSCs) are restricted to development just along the granulocyte pathway. Similarly, acute erythroid leukemia is also an HSC disorder, and the leukemia cells belong to the erythroid lineage only.

The advent of panels of monoclonal antibodies to cell surface markers has led to the description of hematopoietic progenitor cell (HPC) compartments and advanced leukemia sub-typing regarding the cell of origin. Some leukemias are assigned to a lineage-committed HPC within a longstanding branching map for the offspring of HSCs. For example, common pre-B acute lymphoblastic leukemia (ALL) is seen as arising in a B-lineage-committed cell and T-cell acute lymphoblastic leukemia (T-ALL) in a T-lineage-committed cell, whilst CML and acute myeloid leukemias (AML) are still seen as arising in an HSC [3]. However, later studies have revealed an HSC origin for some leukemias that are thought to arise in a lineage-committed HPC. Replicating common pre-B ALL in mice requires HSC-like cells-lacking B-cell markers [4]. Genome-wide analysis identified a multipotent fetal liver HSC as giving rise to immature B-cells in infant B-ALL [5]. The hallmark PML–RARα oncoprotein in acute promyelocytic leukemia is present in patients’ HSCs, indicating an HSC origin [6].

## 2. Lineage-Affiliated Hematopoietic Stem and Progenitor Cells can Still Adopt a Different Pathway

**Figure 1:**
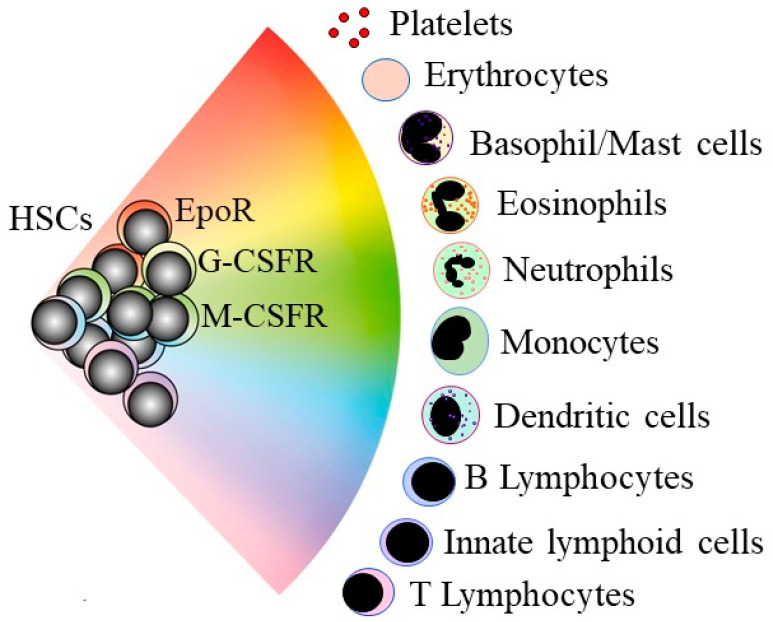
A continuum model for blood cell development. HSCs “choose” a lineage from a continuum of the variety of options. For mouse HSCs, the different colors show that they are a mixture of cells that selectively express the receptors for the lineage-affiliated cytokines erythropoietin (EpoR), granulocyte colony-stimulating factor (G-CSFR), and macrophage colony-stimulating factor (M-CSFR). HSCs and hematopoietic progenitor cells retain enough versatility to “step sideways” into a different pathway.

An understanding of how normal stem cells “choose” to develop towards the end-cell type is clearly important to unravel whether this process is deregulated in CSCs. Classic and long-standing tree-like models depict HSC development as a preferred step-wise and progressive restriction to an end-cell fate via a series of intermediate HPCs. In newer models, HSCs choose a lineage much earlier in a continuum model they “choose” directly from a spectrum of all of the end-cell options (Figure 1) [7]. In keeping with the model, adult human HSCs are mostly a mixture of cells with different lineage-affiliated signatures [8]. Mouse HSCs are a mixture of lineage-affiliated cells that selectively express the cell surface receptors for the lineage-affiliated cytokines erythropoietin (Epo), macrophage colony-stimulating factor (M-CSF), granulocyte colony-stimulating factor (G-CSF), and granulocyte/macrophage colony-stimulating factor (GM-CSF) [9]. The transplantation of single-mouse HSCs that express the megakaryocyte-affiliated von Willebrand factor into an irradiated mouse reveals the existence of HSCs that are biased towards platelet and myeloid development [10]. Surface markers have been used to purify HSCs that are myeloid- and lymphoid-biased, as revealed by transplantation into irradiated mice [11].

There are near-neighbor relationships in the continuum model between the cell lineages (Figure 1), enabling HSCs and HPCs that affiliate to a fate to step-sideways to differentiate towards an end-cell type that does not belong to the initial lineage chosen. Alternative trajectories for mouse HSCs and HPCs were revealed by RNA sequencing of single cells [12]. Mouse HPCs that are biased towards megakaryocyte development can take a “sideways step” to erythropoiesis [13]. HSCs that have progressed in some way along a pathway are still able to divert along another pathway, because T-cell progenitors can give rise to macrophages and natural killer cells [14]. This flexibility is important for a demand for an increased production of, for example, granulocytes or platelets. The hematopoietic cytokines drive their emergency production, and for many years, they were viewed as ensuring the survival and expansion of lineage-committed HPCs. Some are now known to instruct cell lineage. The M-CSF is required for T-cell progenitors to give rise to macrophages [14] and instructs myeloid fate in HSCs [15] and macrophage fate in granulocyte/macrophage HPCs [16]. Epo instructs an erythroid lineage bias in HSCs and HPCs [17]. The G-CSF and the GM-CSF instruct the neutrophil development of granulocyte/macrophage HPCs [16,18].

In addition to producing the different types of cells with a tissue, a cardinal feature of stem cells is that they self-renew and can produce a lifetime supply of cells. A feature of cancers is their unlimited capacity to divide in order to sustain a tumor. This is reminiscent of the self-renewal of stem cells, and therefore, stem cells have been viewed for some time as a primary target of transformation for cancer, as brought to the fore by the cancer stem theory [1]. For cancers arising in a stem cell, it is reasonable to assume that CSCs retain stemness, a capacity to self-renew. There are also no findings to support the notion that an oncogene can convert a progenitor cell that is clearly lineage-committed and lacking self-renewal, particularly an HPC, to the stem cell-like state that is needed to sustain cancer. As to stemness, common networks of proto-oncogenes and tumor suppressors influence normal stem cell self-renewal and survival and the survival and proliferation of cancer cells. For example, the proto-oncogene *Bmi-1* promotes stem cell self-renewal and is required for cancer cell proliferation [19]. Activating *KRAS* mutations drive colorectal cancer and optimize transformation by imposing a stem-like state [20]. *Raf1* plays a role in the survival of HSCs, and for breast and colon cancers, the level of expression is an indicator of the success of chemotherapy [21]. Many known human proto-oncogenes include growth factors, their receptors and signal transducers, transcription factors, and bcl2, a regulator of cell death. Their involvement in regulating the behavior of normal cells and deregulating the behavior of cancer cells is well documented. As following, the focus of this review is a category of oncogenes that appear to restrict the lineage versatility of normal stem cells, so that CSCs give rise to only one type of cell.

## 3. Lmo2-Mediated Lineage Restriction of LSCs

**Figure 2:**
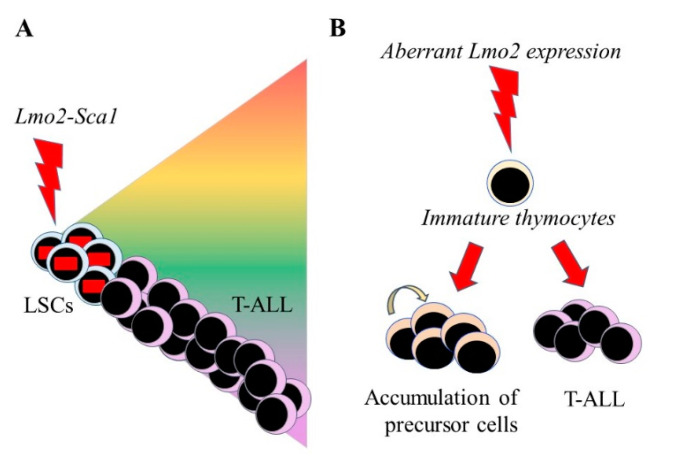
The influence of *Lmo2* expression on hematopoietic stem and progenitor cells (**A**) In transgenic mice, the expression of *Lmo2* is restricted to hematopoietic stem and progenitor cells, via the Sca-1 promotor, leading to T-cell acute lymphoblastic leukemia (T-ALL). The oncogene is active solely within the leukemia stem cells (LSCs) and therefore not essential for the survival/proliferation of the more mature lineage-affiliated leukaemia cells. (**B**) Aberrant expression in immature thymocytes leading to an accumulation of precursor cells and T-ALL.

Some oncogenes deregulate the inherent ability of HSCs and HPCs to divert along another pathway by restricting LSCs to a single cell lineage. The *Lmo2* gene encodes a LIM-only transcription factor that is a frequent target for chromosomal translocation in T-ALL [22]. The expression of the human *Lmo2* oncogene was restricted to HSCs/HPCs in transgenic mice under the control of the stem cell-specific promotor Sca1. The mice developed an aggressive T-ALL with a genetic signature that is analogous to the human lineage-restricted disease (Figure 2). *Lmo2* had functioned in a “hit-and-run” manner, because expression is only maintained in HSCs/HPCs. Further strains of transgenic mice were developed that restrict the Cre-mediated activation of *Lmo2* to allow expression at different stages of B lymphocyte development. They reveal that B-cells are sensitive to transformation by *Lmo2*, and unexpectedly, ectopic expression within committed pro-B-cells and germinal-center B-cells, led to an aggressive T-ALL. *Lmo2* can, therefore, impose a T-cell developmental program on committed B-cells or at least initiate reprogramming into leukemic T-cells and seems to have a set mode of action in provoking leukemia [23].

Does *Lmo2* play a role in T-cell development? From studies of conditional knockout mice, *Lmo2* does not have a mandatory role [24], but knockout mice do not exclude a regulatory role. *Lmo2* is expressed within immature CD4/CD8 double-negative thymocytes and aberrant *Lmo2* expression in committed and immature T-cells in the thymus led to self-renewal, an accumulation of early precursors, and transformation of T-cell precursors to T-ALL (Figure 2) [25]. Whilst we do not know how ectopic *Lmo2* expression imposes a T-cell fate within B-cells in transgenic mice, *Lmo2* can influence the availability of fate options within cells. The transient expression of *Lmo2* together with the five transcription factors Run1t1, Hlf, Prdm5, Pbx1, and Zfp37 confers a multilineage potential onto committed lymphoid and myeloid progenitors and differentiated myeloid (monocyte, macrophage, and granulocyte) cells. The reprogrammed cells are termed induced-HSCs [26].

A caveat to *Lmo2* setting the lineage of LSCs to T-cell development in the transgenic mice is that *Lmo2* might transform an HSC/HPC that has already “chosen” a fate. *Lmo2* expression might therefore enforce a pre-existing lineage disposition rather than setting the fate of a multipotent LSC. In either case, the oncogene appears to ensure that differentiating leukemia cells belong to only one lineage. It is important to bear in mind that HSCs and HPCs have alternative trajectories, with other options remaining latent. That lymphoid leukemia rarely, if ever, diverts into a myeloid leukemia supports the notion that the fate of LSCs is indeed fixed. Additionally, *Lmo2* expression in transgenic mice leads to an aggressive T-ALL, and perhaps, it is unlikely that *Lmo2* expression changes a progenitor cell into a stem cell-like state for cancer.

## 4. BCR-ABLp190- and BCR-ABLp210-Mediated Lineage Restriction of LSCs

Depending on the breakpoint within the *BCR* gene partner, there are two major isoforms of the oncogenic BCR–ABL protein. Leukemia cells from around three quarters of cases of Philadelphia-chromosome-positive B-ALL express BCR–ABLp190, whilst the remaining patients’ cells express BCR-ABLp210 [27]. BCR-ABLp210 is the hallmark of CML, and BCR-ABLp190 is expressed in only 1% to 2% of patients who are a high-risk group [28]. The *c-ABL* proto-oncogene encodes a cytoplasmic tyrosine kinase, and the *BCR*-encoded sequence interferes with the negative regulation of the ABL tyrosine kinase. BCR–ABL is a constitutively active kinase that interacts with cell signaling pathways to deregulate cell behavior.

Restricting the expression of the *BCR–ABLp190* and *BDR–ABLp210* oncogenes in transgenic mice to HSCs, via the Sca1 promotor, leads to human-like B-ALL and CML, respectively [29,30,31]. However, how BCR–ABLp190 and BDR–ABLp210 might set the lineage fate of LSCs and their descendants towards these different pathways is unknown. The two fusion proteins are closely related, but the global phosphorylation analysis of murine pro-B Ba/F3 cells that were engineered to express either BCR–ABLp190 or BDR–ABLp210 has revealed differential signaling through BCR–ABLp190 and BDR–ABLp210. Protein–protein interaction analyses were undertaken for the transfectants of Ba/F3 cells and primary murine multipotent cells. BCR–ABLp190 was observed to interact with cytoplasmic molecules, and BCR–ABLp210 interacted with molecules that were close to the cell membrane. These differences might underlie the differential effects of the two oncogenes in the transgenic mice [32]. BCR–ABLp210 can contribute towards the establishment of a myeloid fate, because forced expression within embryonic stem cells differentiating towards HSCs and HPCs changes the balance of lineage development towards a dominance of myeloid over erythroid fate, indicating that BCR–ABLp210 antagonises erythroid development [33]. However, this does not explain why the leukemia cells in CML belong to only the neutrophil pathway.

## 5. RUNX1-Mediated Lineage Restriction of LSCs

RUNX1 is a transcription factor, and there are different classes of human RUNX1 mutations with each leading to a distinct disease phenotype [34]. Oncogenic RUNX1 proteins arise via either mutation within the DNA-binding or the transactivation domains or translocations that result in fusion proteins. Mutations involving RUNX1 are involved in 14% of AML cases [35]. The inducible expression of different types of RUNX1 fusion oncoproteins in mouse embryonic stem cell-derived blood progenitors influences the development of these cells. The four mutant proteins examine disrupted terminal differentiation, as revealed by colony-forming assays. The two fusion oncoproteins that cause a greater change in gene expression disrupt the balance of lineage priming within progenitors, as revealed by differential chromatin accessibility analysis at promotor elements. RUNX1–EVI1 expression does not cause the loss or gain of a cell lineage, but lineage priming is disrupted whereby chromatin accessibility sites associated with B-cells and megakaryocytes are gained and ones associated with erythroblasts and monocytes are lost. For cells that express RUNX1–ETO, accessibility sites associated with B-cell development and common myeloid progenitors are, gained and ones associated with megakaryocytes are lost [36].

At least two oncogenic “hits” are needed for leukemia. The first oncogene modifies an HSC or HPC, to convert it into a preleukemic (leukemia-initiating) cell. The second insult or perhaps more hits converts the preleukemic cell or a descendant into an LSC that sustains leukemia. In the case of childhood B-ALL, an environmental insult seems to be essential to turn preleukemic cells into leukemia [37]. The need for two hits adds complexity to unravel the role of oncogenes in driving lineage priming or restriction of LSCs. *ETV6*/*RUNX1* can trigger B- and T-cell leukemias. The initiation of *ETV6*/*RUNX1* expression in HSCs and maintenance in their offspring in transgenic mice (*ETV6-^ETV6-RUNX1^* × *Sca1-Cre*) leads to the development T-ALL (34.4%) and B-ALL (6.3%), when the mice are exposed to natural infections in a non-specific pathogen-free environment. The penetrance is low, and perhaps *ETV6/RUNX1* restricts preleukemia cells to the eventual emergence of lymphoid ALL. The second environment event is needed for overt leukemia that also dictates the phenotype. When *ETV6*/*RUNX1* expression is targeted to committed B-cells in transgenic mice, they fail to develop B-ALL even when they are exposed to natural infections or when loss of *Kdm5c*, which is associated with *ETV6*/*RUNX1* B-ALL, is also introduced into committed B-cells. For B-ALL, *ETV6*/*RUNX1* expression and a second event have to occur at the pro-committed B-cell stage of the development with the second event determining cell phenotype [38].

The need for two hits is germane per se to the lineage restriction of LSCs to only one pathway, because perhaps there is a need to close all of the options and ensure the adoption of one. Induced pluripotent stem cells are generated by introducing the four transcription factors, i.e., Oct4, Sox2, Kif4, and c-Myc [39], the generation of these cells is somewhat inefficient, and the generation time was slow. The network that controls and fine-tunes the establishment and maintenance of pluripotency in a stem cell is, therefore, likely to be a more complex interplay of factors. The first “hit” to this network might cascade to destabilise and collapse multipotency, and the second hit facilitate the adoption of one pathway.

RUNX1 influences the lineage trajectory of hematopoietic cells. It plays a key role during the endothelial-to-hematopoiesis transition at the onset of blood cell development [40,41]. RUNX1 binds to chromatin primes for hematopoietic differentiation and represses endothelial cell fate. RUNX1 binding and elevated histone acetylation are essential to the correct pattern of the binding of transcription factors for hematopoiesis, for example, binding of the myeloid/lymphoid-affiliated PU.1 [42,43]. At later stages of hematopoiesis, RUNX1 together with other factors, such as GATA1 (for erythropoiesis), can balance the lineage outcome of HPCs. RUNX1 represses erythroid gene expression during megakaryopoiesis [44], and expression is normally downregulated during erythropoiesis. The RUNX1 DNA-binding (RBD) mutant proteins, which are associated with poorly differentiated AML, disrupt erythropoiesis, when the retroviral vector transduces into murine bone marrow or human cord blood cells, perhaps by antagonizing RUNX1 function [45].

## 6. Oncogene-Mediated Lineage Restriction of Solid Tumors

Ewing’s sarcoma is the second most common malignant bone tumor of children and adults. The reciprocal translocation t11;22 occurs in most cases of Ewing’s sarcoma, giving rise to the oncogenic chimeric protein EWS-FLI-1 [46]. The EWS-FLI-1 protein has the amino terminus of EWS, a member of the TET family of RNA-binding proteins, and the carboxy terminus of Friend leukemia integration 1 transcription factor (FLI-1), a member of the E26 transformation-specific (ETS) family, and is able to bind to DNA in a sequence-specific manner. Ewing’s sarcoma seems to arise from a mesenchymal stem cell or neural crest cell, and EWS-FLI-1 expression in primary human mesenchymal cells triggers a gene expression profile resembling that of Ewing’s sarcoma, therefore recapitulating the initial steps of this disease [47]. EWS-FLI-1 influences cell lineage as expression in the murine myoblast cell line C2C12, which can differentiate into bone, fat, or muscle, blocked myogenesis, and cells expressed the bone marker alkaline phosphatase [48]. FLI-1 has also been shown to influence the lineage trajectories of hematopoietic cells [49]. It is required for HSC development and megakaryocyte commitment and overexpression of FLI-1 in the erythroleukemia-derived cell lines HEL and K562 imparting a megakaryocyte phenotype and inhibiting erythroid differentiation [50]. Similarly, FLI-1 downregulation triggers erythroid progenitors to differentiate into erythrocytes [51]. The level of FLI-1 is therefore important for the choice between megakaryocyte versus erythroid cell lineages.

The transcripts resulting from fusions of the *SYT* (at 18q11) gene with either *SSX1* or *SSX2* (both at Xp11) are diagnostic markers for synovial sarcomas (reviewed in [52]). These sarcomas are also thought to arise in a mesenchymal stem cell, and *SYT–SSX2* reprograms human bone marrow-derived mesenchymal stem cells and myogenic progenitors towards commitment to a pro-neural lineage by targeting neural-specific genes [53]. In addition, whether synovial sarcomas express the SYT–SSX1 or SYT–SSX2 fusion transcript, which are mutually exclusive, influences the nature of the tumor. There are two histological types with monophasic tumors-containing spindle cells, and biphasic tumors are a mixture of spindle cells and epithelial cells that are arranged in glandular structures. Almost all of the tumors containing the *SYT–SSX2* fusion transcript lack gland formation. The SYT–SSX1 fusion transcript is seen in both monophasic and biphasic tumors. Hence, the functional heterogeneity of the two fusion proteins, as related to minor structural differences, is a determinant of the nature synovial sarcoma. [54]. The SYT and SSX proteins do not contain a DNA-binding domain, and SYT-SSX2 interacts with the polycomb repressive complex, leading to the impaired ubiquination of histone H2A and the reactivation of polycomb target genes [55]. An influence on the phenotype of synovial sarcomas is yet unclear.

Mucosa-associated lymphoid tissue (MALT) is classified as a marginal zone B-cell lymphoma. These tumors mostly arise in the stomach (gastric MALT) but can arise in other tissues, for example, in the lung and thyroid (non-gastric MALT). The most common chromosomal abnormality gives rise to a fusion protein comprising of the apoptosis inhibitor AP12 (chromosome 11q21) and the paracaspase MALT1 (chromosome 18q21) [56,57]. Malignant transformation requires at least one more event, and there is longstanding evidence to support co-operation with a chronic infection, such as by *Helicobacter pylori*. [58]. Despite the abundance of neoplastic B-cells in MALT, an HSC/HPC (Sca1^+^ lineage^−^ cells) may be the origin MALT, because the expression of the MALT1 oncogene in these cells recapitulates human lymphoma in mice [59]. In this case, MALT1 has primed HSCs/HPCs toward B-cell development, leading to the accumulation of mature B lymphocytes. MALT1 plays a role in B-cell development, as antibody responses to vaccination are severely impaired in a family with a homozygous MALT mutation [60] and abrogated in MALT1-deficient mice upon immunization [61].

Diffuse large B-cell lymphomas (DLBCLs) are a heterogeneous group of tumors. Most DLBCLs and primarily germinal center types express the human germinal center-associated (HGAL) protein. A Cre-mediated approach was used to express *HGAL* in HSCs/HPCs, pro-B-cells, and germinal-center B-cells, and in each case, the mouse strains developed lymphomas-resembling DLBCLs. The tumors are of the germinal center type, and exon sequencing reveals the presence of the mutations that are seen in human DLBCLs. Hence, the forced expression of HGAL, including within HSCs/HPCs, leads to a B-lineage restricted lymphoma [62]. HGAL is a B-cell-specific adapter protein that enhances B-cell receptor signaling via the activation of Syk, leading to follicular lymphoproliferation [63]. It also controls B-cell motility by modulating the RhoA signaling pathway [64]. Presently, how HGAL might influence the trajectory of HSCs is not known.

## 7. The Epigenome Is Deregulated in Leukemia

Epigenetic controls regulate gene expression within regions of the genome by changing whether they are silent or whether the interaction of genes with transcription regulators is permissive. Changes to the epigenome are inheritable and also influenced by signals that a cell receives from its environment: the landscape, therefore, plays a key role in fine-tuning the gene expression for cell identity. A complex network controls the epigenetic landscape of cells, including the three-dimensional nature of chromatin, epigenetic modifications to DNA without changing its sequence, modifications to histones, and non-coding miRNAs. Further investigation of the *BCR-ABLp210* transgenic mice that develop CML indicated that epigenomic events set the lineage of LSCs towards granulocytes. The reduced-representation bisulfite sequencing profiling of the DNA methylation landscapes for HSCs/HPCs versus LSCs from the *BCR-ABLp210* transgenic mice reveals a global loss of DNA methylation at CpG islands that are methylated at a low-to-moderate level in normal HSCs/HPCs. DNA methyltransferase (Dnmt) 1, which adds methyl groups to DNA, is upregulated in LSCs. Restricting the expression of Dnmt1 to HSCs/HPCs in transgenic mice via Sca1 promotor control phenocopies the *BCR–ABLp210*-provoked hypomethylation changes and leads to a malignancy involving an expansion of granulocytes in the bone marrow and blood [31]. Dnmt1, Dnmt3A, and Dnmt3B interact with EZH2, the catalytic subunit of the polycomb repressive complex 2 [65]. DnmtT1 overexpression leading to the disruption of the association of other Dnmts with EZH2 may explain the hypomethylation within LSCs. Interference with the balance of de novo methylation might lead to *BCR–ABLp210* LSCs adopting a granulocyte trajectory. Disruption to de novo methylation might collapse the network for multipotency, leaving only one and/or enforced one option.

In the *BCR–ABLp210* transgenic mice, the lineage of LSCs, together with their offspring, is set towards granulocytes, even though expression is restricted to LSCs. In this regard, the *BCR–ABLp210* upregulation of Dnmt1 expression is a lasting change, as hypomethylation is conserved in the mature offspring of LSCs [31]. DNA methylation changes are also maintained through cellular division [66]. From all of the above, oncogene-mediated epigenetic re-programming seems to be sufficient for the development of CML. Presently, it is unclear how a perturbation to DNA methylation alters gene expression for HSC/HPC transformation and the lineage restriction seen in CML.

In keeping with the above upregulation of Dnmt1, Dnmt1 is significantly overexpressed in AML and myelodysplastic syndromes (MDS) [67]. More than 90 miRNAs are predicted to target Dnmt1 and some of these are deregulated in AML and MDS. For example, miR-495 is downregulated in AML cases bearing mixed lineage leukemia (MLL) rearrangements and functions as a tumor suppressor [68]. Dnmt1 may be involved at the pre-leukemic stage of myeloid leukemias, as it is needed for the maintenance of HSCs and HPCs which are decreased in a zebrafish mutant cell line that has a stop codon mutation in Dnmt1 [69]. Dnmt1 also plays a role in carcinomas, as knockdown reduces the number of cancer-initiating cells and CSCs in the case of colon and breast cancer [70].

Dnmt2 plays a proposed role in myeloid leukemia, as it is expressed at a high level in the CML-derived K562 cell line, methylates tRNA, and azacytidine, which is approved for the treatment of MDS, inhibits RNA methylation at Dnmt2 target sites [71]. Whist Dnmt1 mutations are rare, Dnmt3A mutations are frequently observed in AML and MDS, and Dnmt3A and Dnmt3B play a role in acute AML (reviewed in [72]). For CML, Dnmt3A mutations appear to be important for the clonal evolution of CML, because they are found in Philadelphia-chromosome-positive and Philadelphia-negative clones of patients [73]. Acute promyelocytic leukemia, which is classified as AML M3, is characterized by a translocation that leads to the creation of a fusion between the *PML* and *RARA* (retinoic acid receptor) genes. Dnmt3A is required for *PML–RARA* to initiate acute promyelocytic leukemia, as it is needed for *PML–RARA* to drive the aberrant self-renewal of mouse bone marrow cells ex vivo [74]. The PML–RAR fusion protein is able to recruit Dnmt1 and Dnmt3A that are otherwise dispersed in the nucleus to target promotors, such as RARb2, and induce hypermethylation [75].

The epigenetic landscape is also perturbed in myeloid leukemia at the level of post-translational modifications to histones. Acetylated histone H3 at lysine 27 (H3K27ac) and trimethylated histone H3 at lysine 4 (H3K4me3) are highly enriched at active promoters, which correlate with transcription. Chromatin silencing is associated with modifications such as trimethylation of histone H3 at lysine 9 (H3K9me3) and at lysine 27 (H3K27me3). EZH2 is the catalyst to H3 methylation at lysine 27, and *EZH2* mutations are common in patients with MDS and myeloproliferative neoplasms (12%). They result in chain termination or abrogation of histone methyltransferase activity, and *EZH2* has been proposed as a tumor suppressor for myeloid leukemias [76]. Histone deacetylase (HDAC) 5 and HDAC7 are often overexpressed in AML, CML, and ALL, and a low level of expression of HDAC4 is common in these malignancies (reviewed in [77]). Accordingly, HDAC inhibitors, such as valproic acid, have been used in clinical trials in combination with other anticancer agents for differentiation therapy of AML [78]. Non-coding miRNAs, which play a role in the recruitment of polycomb repressive complex 2 to target genes, have also been identified as playing a role in the development of AML [79]. Subtypes of AML have distinct miRNA profiles; for example, the oncogenic miRNA miR-9 is overexpressed in AML cases bearing MLL rearrangements. A proposal is that miRNAs collaborate with known oncogenes or tumor suppressors, and the profiles of miRNAs corelate with disease phenotype and prognosis [80]. From the above findings for leukemia cells and comparable findings for solid tumors, perturbation to the epigenome is important to the anarchic behavior of cancer cells.

## 8. Epigenetic Control of Stem Cell Development

It is well-established that epigenetic processes play a crucial role in normal stem cell development by fine-tuning the expression of genes, as highlighted by findings from studies of embryonic development. This topic has been reviewed in detail elsewhere [81]. DNA methylation is a major player, because methylated genes are transcriptionally inactive. During embryonic development there is genome-wide demethylation for pluripotency. Post-translational modifications to histones also regulate the epigenetic status of stem cells. Embryonic stem cells have an abundance of the modifications that are associated with transcriptionally permissive chromatin. Recent studies have highlighted that the regain of H3K27ac for active transcription is important for cells to re-establish a program, as they exit from mitosis, and therefore, to lineage identity [82]. miRNAs also regulate the lineage status of stem cells by modulating their pluripotency and differentiation [83]. They are also important for the establishment and maintenance of embryonal stem cell identity and their differentiation [84].

A long-standing notion from studies of HSCs is that there is the low-level priming of expression of lineage-affiliated genes. Some investigators have argued that there is order of the priming of genes for hematopoiesis whereby HSCs first prime or express megakaryocyte- and erythroid-associated genes and that a megakaryocyte-biased HSC sits at the apex of hematopoietic cell development [9]. Lymphoid genes are transcribed later. The regulation of the activation of the *Cd19* gene for B-cell development has been investigated, and epigenetic priming occurs before gene expression for lineage specification. Chromatin remodelling, as revealed by the formation of DNase I hypersensitive sites (DHS foot-printing), the presence of histone marks, and the possible removal of CpG methylation at the promotor are all important. As revealed by DHS foot-printing, the upstream enhancer to *Cd19* is remodelled first, and this occurs in multipotent HSCs/HPCs. In these cells and in the absence of PAX5, the *Cd19* enhancer is remodelled as to having a low level of monomethylation of histone H3 at lysine 9 (H3K9me1). The transcription factor E2A then binds to the enhancer followed by the transcription factors EBF and PAX5. The *Cd19* promoter binds PAX5, and this results in the activation of mRNA transcription. Hence, the enhancer is active before the start of mRNA transcription. Bisulphite sequencing, to measure the level of methylation at CpGs, reveals that the level of methylation at the promotor correlates inversely with the level of gene expression. There is a storage of RNA polymerase II at the enhanced prior to at the promotor, which may also have a bearing on the level of CD19 expression [85].

As considered above, some of the hematopoietic cytokines are known to instruct the lineage of HSCs and HPCs. Hence, how signals from these cytokines translate to transcriptional control of cell type is clearly an important question. From studies of human embryonic stem cell-derived gut endodermal lineage intermediates, investigators have proposed that the poised state of enhancers bookmarks a cell’s identity and that this also allows cells to respond correctly to differentiation cues from the environment. For the endodermal lineage intermediates, differentiation competence at enhancers was shown by mapping histone modifications during pancreatic differentiation. Moreover, prior to any lineage specification, the ability of cells to respond to inductive signals correlates with the poised state of enhancers. Competent enhancers are recognised by the pioneer transcription factors FOXA1 and FOXA2 and subsequently by lineage-inductive factors. Environmental signals may well govern transcription factors binding to their target sequence by means of poised enhancers [86].

A focus on the epigenetic shaping of the gene expression for the lineage identity of cells is a return to Waddington’s epigenetic landscape model, which was proposed in 1957 [87]. It depicts bifurcating valleys that developing cells roll down towards a fate with ridges to the hills of the valleys, maintaining a chosen fate. In Waddington’s model, the epigenetic landscape dictates the hills and valleys and, in turn, modifies the loci that control the expression of the transcription factors that are key to stem-cell decision-making. They include ELF5, OCT4, and NANOG as highlighted by studies of the control of the establishment of the ectoderm, endoderm, and mesoderm lineages of the early embryo [81]. DNA methylation seems to establish the barriers/hills between cell lineages. This is the case for the first decision during embryonic development, namely blastocyst cells choosing to develop towards either embryonic or trophoblast lineages. Preventing Dnmt3a and Dnmt3b de novo DNA methylation in murine embryonic stem cells by knockout allows these cells to adopt an “unwanted” trajectory, as they generate trophoblast cells [88].

## 9. Concluding Remarks

The epigenome has been described as “the judge, jury, and executioner” to the conduct of normal stem cell development [89]. There is evidence to support this view regarding the barriers to the lineage options of stem cells and the integration of the environmental signals that are important for cell fate. Normal stem cell development is deregulated in cancer, and perhaps, it is not too surprising that the control of the epigenetic status of cells is vulnerable to deregulation by oncogenes. Specific and consistent genetic insults are associated with some cancer cell phenotypes. For example, *BCR–ABLp210* is the hallmark of CML, whereby leukemic cells are granulocytes and the targeting of this phenotype-affiliated oncogene to a stem cell in transgenic mice recapitulates human lineage-restricted disease. In this case, the stem/progenitor cells lose their inherent versatility. It is important to note that for LSCs from the *BCR–ABLp210* transgenic mice that DNA methylation at CpG islands is deregulated and that this is sufficient to give rise to leukemia. The epigenetic landscape of patients’ leukemia cells is also perturbed regarding the control of histone modifications and at the level of expression of miRNAs.

Presently, we do not have a clear picture of how the interplay between modulation of the epigenetic landscape at enhancers and promoters controls the availability of lineage options in stem cells and lineage specification. Hundreds of thousands of enhancers throughout the genome outnumber the 20,000 protein-encoding genes of the human genome. Enhancers act at a distance from their promoter to increase the transcription of genes, and their activity can be restricted to a tissue or a cell type. They are first remodelled, active before the start of RNA transcription from target genes, and appear to influence cell responsiveness to environmental cues. Enhancers have been prosed as a central platform that integrates the epigenetic status of cells to a network of lineage transcription factors [90]. Promoter activity is clearly important for full transcription. In this case, do enhancers mastermind the extent of lineage versatility and some oncogenes set cell lineage at promoters? It remains to be seen whether the deregulation of the inherent versatility of stem and progenitor cells for restricting the progeny of CSCs to only one lineage is central to how some oncogenes drive the development of cancer.

## Data Availability

Not applicable.
